# A Guide to Examination of Urine

**Published:** 1886-06

**Authors:** 


A GUIDE TO THE PRACTICAL EXAMINATION OF URINE. *For the use of physicians and students. By* JAMES TYSON, M. D., *Professor of General Pathology and Morbid Anatomy in the University of Pennsylvania; one of the Physicians to the Philadelphia Hospital; Fellow of the College of Physicians of Philadelphia, etc., etc., etc. Fifth edition. Revised and corrected. With colored plates and wood engravings. Pp. XII, 249.*

That the profession has called for five editions of this manual within a comparatively short time is evidence of its value. Within the last few years much has been added to our knowledge of the chemistry of the urine. Facts have been observed and theories propounded without number, and like other rapidly advancing sciences our knowledge is at the same time more than we can use and less than we desire.

From this mass of new material Professor Tyson has carefully selected the most useful portions, and those which have stood the test of experience. The new tests for albumen, and the increased importance of the peptones and mucin receive full consideration.

The work is not a treatise on the chemistry of the urine, but as a working manual for the office and laboratory it has no superior. H. N. M.

---

THE FIELD AND LIMITATION OF THE OPERATIVE SURGERY OF THE HUMAN BRAIN. *By* JOHN B. ROBERTS A. M., M. D., *Professor of anatomy and surgery in the Philadelphia Polyclinic, surgeon to St. Mary's Hospital. Large octavo, pp. 80. Philadelphia:* P. BLAKISTON, SON & CO. *Chicago:* W. T. KEENER.

This essay is a reprint of a paper presented to the American Surgical Association at the 1885 meeting. To those not having a copy of the transactions of the Association, we would say the work contains a complete epitome of the present status of cerebral surgery. It is to be regretted that the valuable discussion which followed the reading of this paper is not appended to the reprint.

